# Cost-effectiveness analysis of procalcitonin and lung ultrasonography guided antibiotic prescriptions in primary care

**DOI:** 10.1007/s10198-024-01694-y

**Published:** 2024-05-18

**Authors:** Giulio Cisco, Armando N. Meier, Nicolas Senn, Yolanda Mueller, Andreas Kronenberg, Isabella Locatelli, José Knüsli, Loïc Lhopitallier, Noemie Boillat-Blanco, Joachim Marti

**Affiliations:** 1 Centre for Primary Care and Public Health (Unisanté), University of Lausanne, Lausanne, Switzerland; 2 Institute for Infectious Diseases, University of Bern, Bern, Switzerland; 3 Lausanne University Hospital, University of Lausanne, Lausanne, Switzerland; 4 Gare10 Lausanne General Practice, Lausanne, Switzerland

**Keywords:** Procalcitonin, Lung ultrasonograph, Cost-effectiveness analysis, Antibiotic resistance, D610, I100, H430

## Abstract

Antimicrobial resistance comes with high morbidity and mortality burden, and ultimately high impact on healthcare and social costs. Efficient strategies are needed to limit antibiotic overuse. This paper investigates the cost-effectiveness of testing patients with lower respiratory tract infection with procalcitonin, either at the point-of-care only or combined with lung ultrasonography. These diagnostic tools help detect the presence of bacterial pneumonia, guiding prescription decisions. The clinical responses of these strategies were studied in the primary care setting. Evidence is needed on their cost-effectiveness. We used data from a cluster-randomized bi-centric clinical trial conducted in Switzerland and estimated patient-level costs using data on resource use to which we applied Swiss tariffs. Combining the incremental costs of the two strategies and the reduction in the 28-days antibiotic prescription rate (APR) compared to usual care, we calculated Incremental Cost-Effectiveness Ratios (ICER). We also used the Cost-Effectiveness Acceptability Curve as an analytical decision-making tool. The robustness of the findings is ensured by Probabilistic Sensitivity Analysis and scenario analysis. In the base case scenario, the ICER compared to usual care is $2.3 per percentage point (pp) reduction in APR for the procalcitonin group, and $4.4 for procalcitonin-ultrasound combined. Furthermore, we found that for a willingness to pay per patient of more than $2 per pp reduction in the APR, procalcitonin is the strategy with the highest probability to be cost-effective. Our findings suggest that testing patients with respiratory symptoms with procalcitonin to guide antibiotic prescription in the primary care setting represents good value for money.

## Key Points

**Table:** 

1. While most patients who present to their general practitioner with lower respiratory tract infections receive a prescription for antibiotics, only a few of them have bacterial community-acquired pneumonia requiring antibiotics. Testing patients with procalcitonin alone or with procalcitonin combined with a lung ultrasonography may reduce the number of unnecessary prescriptions (Lhopitallier et al. 2021).
2. With this study, we try to find out whether these two diagnostic strategies are cost-effective compared to the standard of care, by applying socio-economic evaluation techniques in the health sector, such as the calculation of incremental cost-effectiveness ratios and the cost-effectiveness acceptability curve. Furthermore, for the first time, there was an attempt to include in the analysis a monetary value to be attributed to the risk of developing greater resistance because of the prescriptions.
3. The study showed that the number of prescriptions decreases significantly with procalcitonin testing at moderate incremental costs. On the other hand, the additional lung ultrasonography results in considerable additional costs but no significant improvement in effectiveness.

## Introduction

Lower respiratory tract infection (LRTI) is one of the most common reasons for consulting primary care physicians (PCPs) and a reason for inadequate antibiotics prescribing [1–4]. While most patients presenting with LRTI to their PCP receive an antibiotic prescription, only 5 to 12% of them have bacterial community-acquired pneumonia requiring antibiotics [5]. The absence of distinct symptoms to differentiate community-acquired pneumonia of bacterial, versus viral, origin makes identification of these patients difficult [6].

Diagnostic strategies are available to improve the appropriateness of antibiotics prescriptions. Procalcitonin, a biomarker that is higher in bacterial pneumonia compared with viral acute respiratory infections [7], has recently become available as a point-of-care test. Another available tool is point-of-care lung ultrasonography, used to detect lung consolidation and to confirm the diagnosis of pneumonia [8–10]. In a recent study conducted in Switzerland [11, 12], it was shown that point-of-care procalcitonin testing led to an absolute reduction of 26 percentage points (pp) in the antibiotic prescription rate (APR) compared to usual care (0.43 vs. 0.69, p-value = 0.001), whereas there was no significant difference when adding lung ultrasound to procalcitonin (0.43 vs. 0.39, p-value = 0.709). The difference in APR between usual care and procalcitonin testing and lung ultrasound combined was significant (0.39 vs. 0.69, p-value = 0.001). For secondary outcomes, there were no differences between the three groups regarding clinical failure by day 7 and serious adverse outcomes by day 28. The data from this study were the basis for the analysis we present below.

While this evidence suggests that procalcitonin point-of-care testing in primary care can reduce inappropriate antibiotic use, it is unclear whether it is cost-effective. Studies have recently shown promising results in terms of cost savings and cost-effectiveness of procalcitonin testing, but in different settings and patient groups, such as patients with critical infections or patients hospitalized with respiratory tract infections [13, 14], with suspected sepsis [15, 16], or for infants [17, 18]. One study evaluated the cost-effectiveness of procalcitonin-guided antibiotics for the management of patients with LRTI in primary care but based on procalcitonin measured in a central laboratory [19]. In this study, we use trial data to conduct an economic evaluation of point-of-care procalcitonin testing only as well as combined with lung ultrasonography in primary care. More specifically, the main objective of the study is to evaluate the cost-effectiveness of algorithms utilizing procalcitonin alone and in combination with lung ultrasound to reduce antibiotic prescribing among adult patients with LRTI, managed in primary care settings in Switzerland, in comparison to standard care practices.

## Methods

### Trial design

Our analysis is based on data from a three-arm, pragmatic, cluster-randomized clinical trial conducted in primary care practices in western Switzerland from September 2018 to March 2020. Consecutive patients aged 18 or older with clinical pneumonia were included by PCPs and managed using different strategies. 60 PCPs were randomized in a 1:1:1 ratio to a study group to decide on antibiotic prescription either guided by: sequential procalcitonin and lung ultrasound point-of-care tests (UltraPro group); point-of-care procalcitonin only; or usual care. The interventions in the three arms were as follows:

- UltraPro: the UltraPro algorithm was employed, which integrates the point-of-care procalcitonin test with lung ultrasound to guide antibiotic prescription decisions. If the procalcitonin test shows elevated levels (≥ 0.25 μg/L) and ultrasound detects lung consolidation, antibiotics are recommended. The choice of antibiotic and additional diagnostic tests is at the discretion of the PCP.
- Procalcitonin: procalcitonin test is used to guide antibiotic prescription decisions. If the procalcitonin test indicates elevated levels, antibiotics are recommended and the PCP determines the type, dosage and duration of treatment, and any additional tests.
- Usual care: in this arm, patients receive standard care without any specific algorithmic guidance. PCPs manage patients according to their routine practices without using one of the two tests to guide antibiotic prescriptions.

Patient follow-up was done by phone interview on days 7 and 28, and by a self-reported patient diary on symptoms.

In total, 469 participants were included in the trial (152 in UltraPro group, 195 in the procalcitonin group and 122 in usual care group), of which 435 participants (93%) had complete follow-up. All patients assigned to each intervention group received the intended intervention. Full descriptions of the study design and the characteristics of study participants are published elsewhere [11, 12]. The Swiss ethics committee of the cantons of Vaud and Bern approved the protocol (2017–01246). All study participants gave their written consent. An external independent monitoring board supervised the trial.

### Cost-effectiveness analysis

We applied cost-effectiveness analyses (CEA) following best practices in health economics [20, 21] to estimate the incremental cost for each pp reduction in antibiotic prescription, comparing the two interventions with usual care. The perspective applied is the societal one, since in addition to healthcare costs we also include costs due to productivity loss and antibiotic resistance. Results are expressed in terms of incremental cost-effectiveness ratios (ICER). To investigate which strategy is the most desirable given different levels of willingness to pay (WTP) to reduce antibiotic prescriptions in a health care system, we also studied the cost-effectiveness acceptability curve (CEAC) applying a probabilistic sensitivity analysis (PSA), using the ‘dampack’ library in R version 4.1.3.

### Model parameters

We used the trial primary outcome, i.e. 28 days APR, as our effectiveness indicator [12].

We applied a combination of bottom-up and top-down approaches to estimate costs. Eight cost categories were considered, whose magnitudes depended on the volume (use of resources) revealed in the study and the unit prices derived from the Swiss literature and tariffs:

- Training costs: PCPs, medical instructors, and PCP assistants received additional training. This cost was calculated by multiplying the opportunity cost of working time (based on salary) by the number of training hours [22, 23].
- Product costs: purchase and maintenance costs of devices used for diagnostic testing.
- Initial consultation costs: these costs include the time taken by PCPs for examinations and the costs of performed tests considering standardized Swiss medical tariffs [22, 24].
- Cost of antibiotic prescription: this cost is calculated considering the amount and typology of antibiotic prescriptions observed in the trial and combining them with the relative unit prices [25].
- Cost of other drug prescriptions: this cost is calculated considering all the other medications and their unit price [25].
- Costs for healthcare treatment: this category includes costs for chest X-rays, hospitalization, and repeated PCP visits recorded during the trial. We considered reimbursement tariffs and standardized medical prices [24, 26, 27].
- Productivity loss: these costs are calculated considering the sickness duration from trial data and assuming that the patients earn the median Swiss wage. A one-third drop in productivity during restrictions is also assumed [28].
- Antibiotic resistance cost: The estimated indirect cost due to antibiotic resistance is calculated considering the overall estimated antibiotic resistance costs in the European Union, divided by the total number of prescriptions. The value, converted to CHF (Swiss Franc) and adjusted for inflation, is added to patients with at least one prescription [29–42].

A more detailed description of the cost calculation can be found in the appendix. Since the duration of the follow-up was 28 days, we did not apply any discount rate.

### Sensitivity analysis

We conducted a PSA to account for parameter uncertainty. In this way, the variability of costs and effectiveness recorded in the patient level-data are taken into account in the analysis. A simulation with 10′000 random draws was run, by combining the iterations of costs and effectiveness. We assumed a Gamma distribution for the cost and a Beta distribution for the APR.

With these inputs, the CEAC shows the probability that each of the three interventions is the most cost-effective when varying the WTP [43], which in this case is the amount that policy-makers are willing to pay to reduce APR by one percentage point. The analysis therefore shows which strategy policy-makers should take given their willingness to spend a certain amount of money to reduce antibiotic prescriptions.

To consider possible sources of uncertainty and to verify the robustness of our findings, we considered four different scenarios in which we tested the impact of possible deviations in cost and prescriptions accounting. This analysis and its results are detailed in the Appendix.

## Results

### Base case analysis

The average costs per patient, excluding only hospitalisations, were: CHF 634 (95% CI 582 to 686) in the UltraPro group (procalcitonin + ultrasound), CHF 567 (95% CI 518 to 616) in the procalcitonin group and CHF 509 (95% CI 455 to 563) in the usual care group (Table 1). (Consider an exchange rate where 1 CHF = 1.045 US dollars).

**Table 1:** Mean costs (95% CI) in the three study groups, expressed in 2022 CHF (1 CHF = 1.045 $)

Cost category	Usual care	Procalcitonin	UltraPro
Training	20.84 (−)	36.96 (−)	78.64 (−)
Product	0 (−)	10.61 (−)	20.72 (−)
Initial consultation	89.76 (87.15 to 92.37)	112.39 (110.35 to 114.43)	112.95 (110.34 to 115.56)
Antibiotics prescription	26.53 (21.96 to 31.10)	12.37 (9.27 to 15.47)	12.94 (9.53 to 16.35)
Other drugs prescription	12.95 (11.11 to 14.79)	17.92 (16.40 to 19.44)	20.80 (18.94 to 22.67)
Healthcare	31.11 (25.92 to 36.30)	23.59 (19.65 to 27.53)	22.13 (18.09 to 26.17)
Productivity loss	321.95 (270.33 to 373.58)	349.72 (302.60 to 396.84)	362.86 (313.15 to 412.57)
Antibiotic resistance	5.71 (5.05 to 6.37)	3.24 (2.68 to 3.80)	3.30 (2.67 to 3.93)
Total	508.85 (454.72 to 562.98)	566.80 (517.90 to 615.70)	634.35 (582.22 to 686.48)

For the Training and Product categories, there is no CI since the respective values are fixed within each category and arm, as calculated using a top-down approach, i.e. there is no variability between different individuals

**Fig. 1:**
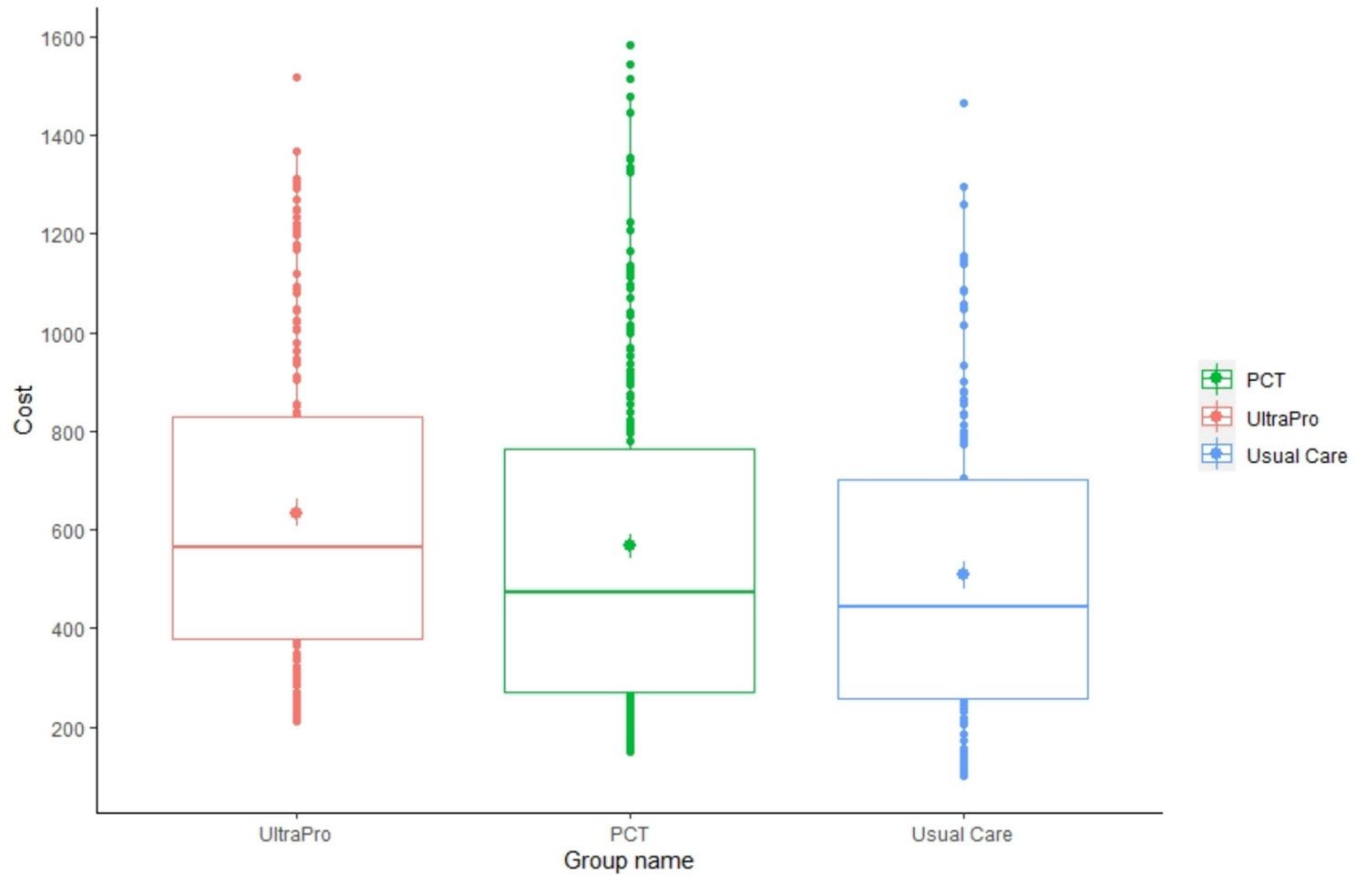
Distribution of total cost per patient in the three arms—base case scenario. Within boxplots, the mean is represented by a dot, and the 25th percentile, the median, and the 75th percentile by lines

UltraPro patients incurred higher costs than patients in the usual care group (p = 0.001), but there was no difference between the procalcitonin and usual care groups (p = 0.132) (Fig. 1). The difference between the UltraPro and procalcitonin groups seems to show some divergence, although it does not reach statistical significance (p = 0.066).

The ICERs are respectively CHF 4.2 (1.5 to 10.8) ($4.4) per pp reduction in antibiotic prescription comparing UltraPro to usual care, and CHF 2.2 (− 0.7 to 7.6) ($2.3) for procalcitonin versus usual care (see Table 2).

**Table 2:** Incremental cost-effectiveness

Groups comparison	Incremental cost, 2022 CHF (95% CI)	APR reduction, percentage points (95% CI)	ICER (95% CI calculated with Fieller method), CHF/percentage points reduction
UltraPro vs usual	126 (49 to 202)	30 (12 to 46)	4.2 (1.5 to 10.8)
Procalcitonin vs usual	58 (− 18 to 133)	26 (10 to 41)	2.2 (− 0.7 to 7.6)

**Fig. 2:**
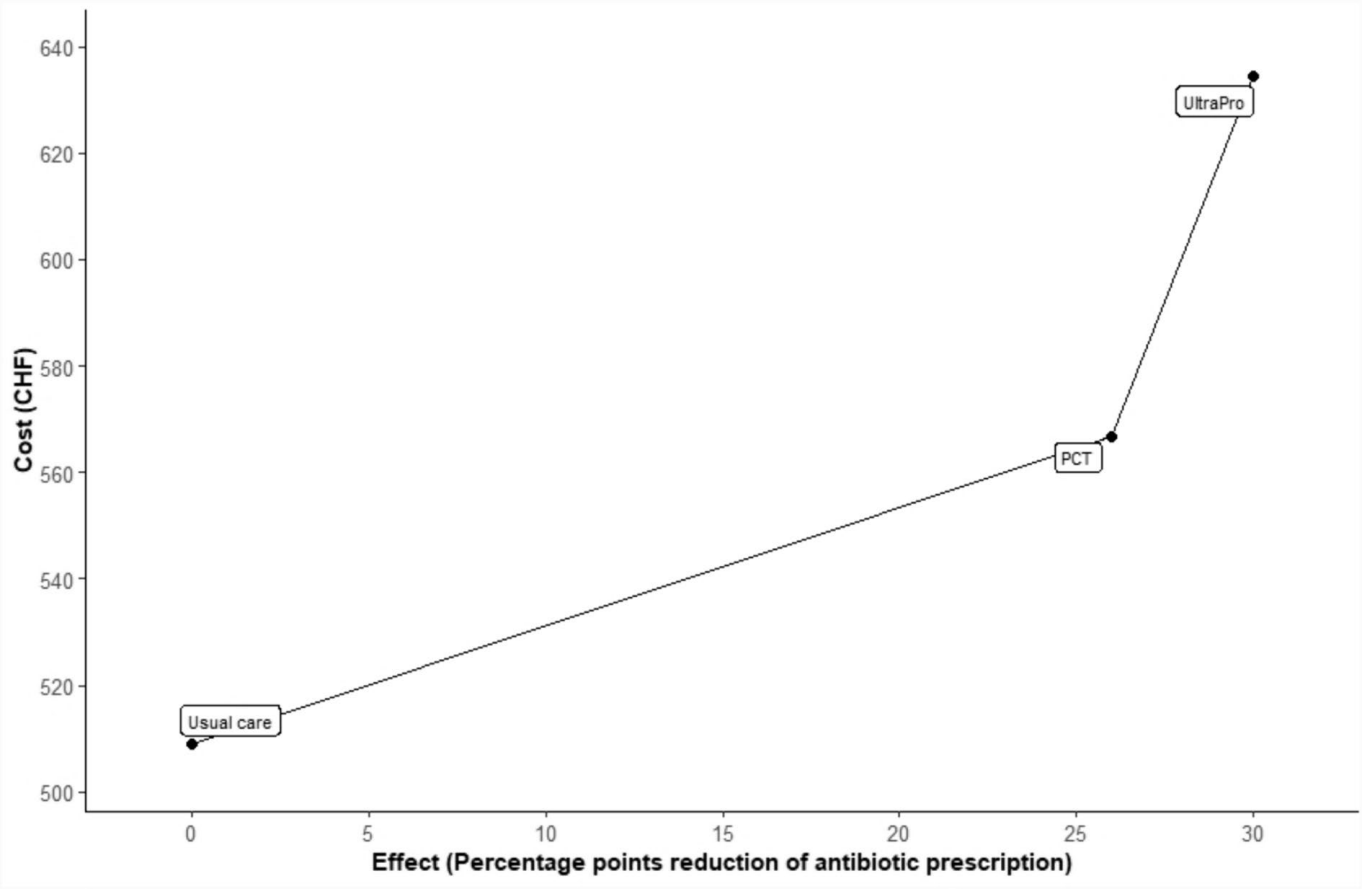
Incremental cost-effectiveness ratio—base case

The incremental cost-effectiveness ratios of the three strategies are compared in Fig. 2.

### Probabilistic sensitivity analysis

**Fig. 3:**
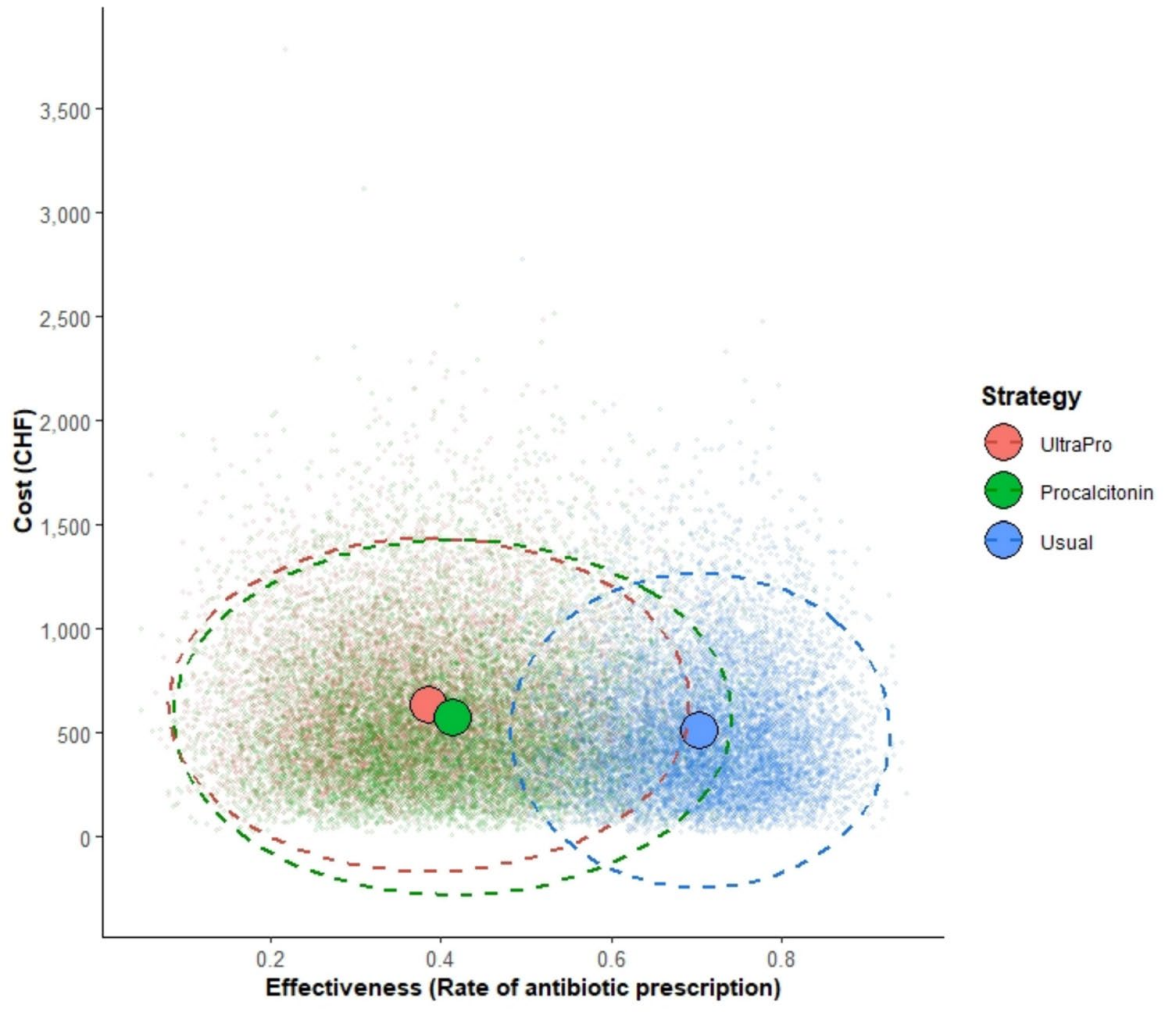
Probabilistic sensitivity analysis—base case. The means and the 95% confidence ellipse after 10,000 replications are represented, respectively, by the large dots and the dotted lines

**Fig. 4:**
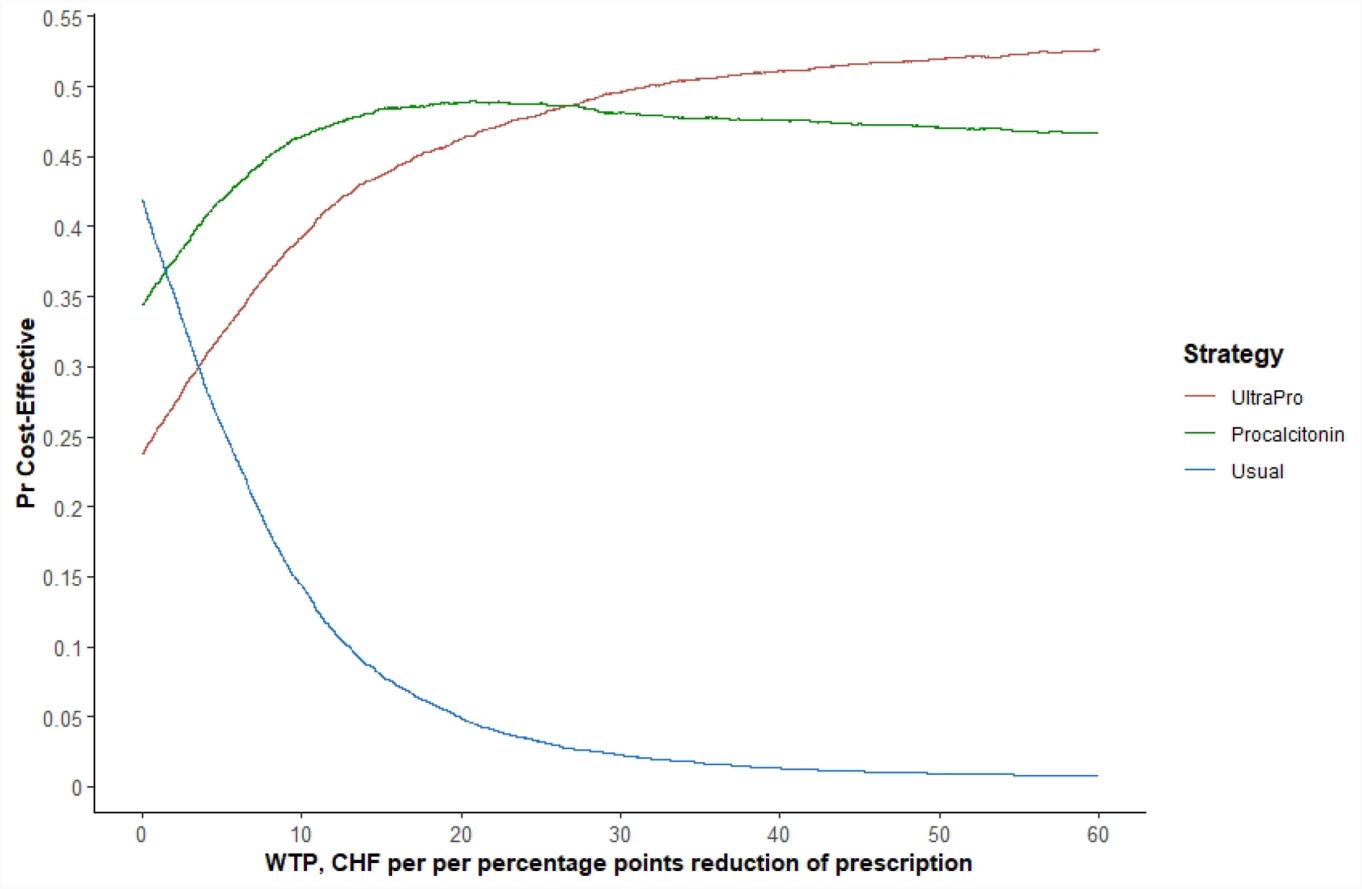
Cost-Effectiveness acceptability curve—base case

Based on the distributions indicated above, we conducted the PSA (Fig. 3) and plotted the CEAC (Fig. 4).

For very low values of WTP – between CHF 0 and CHF 2 ($0 and $2) per pp reduction in the APR—usual care strategy maximises the average expected benefit. For a WTP between approximately CHF 2 and CHF 25 ($0 and $26), procalcitonin is the favoured choice, whereas for very high values (WTP greater than CHF 25) UltraPro becomes the preferred option. This means that, even for a rather low WTP (even about CHF 2 per point of prescription reduction), procalcitonin point-of-care testing has the highest probability of being the best strategy, namely the one that maximises the net monetary benefits.

Overall, the cost-effectiveness per pp reduction in prescriptions is robust considering the different scenarios (see appendix).

## Discussion

Our findings suggest that a LRTI managed with procalcitonin point-of-care testing in primary care does not have statistically significantly different costs from a patient treated with standard usual care. However, APR is much lower after procalcitonin testing compared to usual care [12]. In contrast, using ultrasonography as an additional tool after procalcitonin (UltraPro) markedly raises costs but does not reduce antibiotic prescriptions further, making this option less attractive.

Nevertheless, as unlike other indicators—such as dollars per QALY gained—for which there are thresholds from the literature or national guidelines, which determine whether a project is considered acceptable or not, there is no official threshold expressed in “dollars per pp reduction in APR”. In other words, it is unclear to what extent CHF 2.2 per percentage point of APR reduction represents good value for money. However, we can compare this to the results from literature. For example, for patients with LRTI, point-of-care C-reactive protein testing costs £9.31 ($11) per antibiotic prescription avoided [44]. In the context of acute respiratory tract infections in the US, the WTP was $43 (range: 0–333) per antibiotic prescription safely avoided [19].

Our analysis indicates which option has the highest probability of being the most cost-effective as WTP values vary (Fig. 4): the strategy with the highest probability of being cost-effective if WTP is close to zero is usual care. If a range of higher WTP values is considered, the procalcitonin test becomes the most cost-effective. UltraPro becomes the first option when WTP is higher (i.e. above CHF 26 per pp reduction). Considering the ranges and uncertainty of WTP for pp reduction in the literature, we consider the procalcitonin strategy to be the recommended one.

Our ICERs show cost-effectiveness at the margin. Expressed in absolute terms, if the probability of each patient receiving an antibiotic prescription through the procalcitonin strategy were to be lowered by 26 pp, it would cost approximately $60 per patient. To get an estimate of the effect on the total budget, this amount should be multiplied by the total number of LRTI patients in Switzerland, which we roughly estimate at 200,000–250,000 per year.

However, taking an antibiotic is not in itself bad behaviour. It becomes so if unnecessary antibiotics are prescribed, because this may cause future burdens to the patient and society. Procalcitonin, with or without lung ultrasonography, seem to be useful tools for reducing prescriptions. Procalcitonin alone would seem to allow this at a relatively lower expense than combined with ultrasonography.

### Accounting for resistance

The reason for limiting antibiotic consumption is the risk of developing resistant strains, which are difficult for the healthcare system to manage. Although our estimate of resistance cost is consistent with previous research [38], we acknowledge that a more precise estimation method would make our analysis more accurate. While the results in this study do not appear to rely heavily on resistance costs, we believed it was better to include them than ignore them at all. Furthermore, the macro impact of resistance costs on a country’s healthcare system is quite large and there is a lot of uncertainty about the cost estimation of antibiotic resistance, especially for the US [45–48]. If scenarios with significant resistance costs emerge in the future, UltraPro and procalcitonin diagnostic tools will be more cost-effective and could dominate the usual care strategy (i.e. improve outcomes and reduce costs).

### Strengths

Although Swiss parameters are used for cost estimation, good external validity is expected because the ICER captures the relative difference between the interventions studied and usual care. Although the total costs of treatment and control groups would vary with other country-specific values, the relative differences should not be large.

An attempt was also made to include resistance costs, an innovative aspect in this type of CEA.

On a methodological level, costs were estimated according to precise guidelines and tariffs and the inclusion of the PSA also allowed for uncertainty to be considered. Moreover, the construction of various scenarios helped to show the robustness of the findings.

Keeping in mind that resistance costs are likely to be underestimated and that economies of scale for new diagnostic methods may reduce procalcitonin testing costs, it can probably be said that procalcitonin point-of-care testing will become even more attractive over time.

### Limitations

First, estimation of resistance costs is quite rough, due to the lack of sufficient data for a more precise assessment.

Also, we conducted this analysis in a static context, as the time horizon considered for measuring the number of prescriptions was 28 days. In fact, no discounting was applied. We treated the incurrence of costs and effects at the initial time. A possible argument against this approach is that the costs and effects associated with ABR do not arise at the present time, but rather incur in the future. Once more precise estimates of these costs become available, analysis with an extended time horizon will be possible.

Finally, normally in health economics evaluation it is preferable to use a metric that captures the status of the patient, such as Quality-Adjusted Life Years (QALY), in order to obtain an incremental Cost-Utility Ratio, which can be convenient for comparing performance with a threshold of acceptability in terms of money per QALY. However, in our case we thought that the indicator we used, the APR, which is more specific and context-sensitive, was better suited to capture the effects. In fact, over the time horizon considered, we would not expect substantial variation in QALYs between groups, because people presented to the doctor sick and with similar pneumonia symptoms. Our choice is also consistent with recommendations in the literature, where it is emphasised that since procalcitonin testing reduces antibiotic use without altering symptom duration, hospitalisation risk, or mortality, assessing the effectiveness of procalcitonin in terms of QALYs would not capture the value of procalcitonin testing [19].

### Recommendations for further research

In order to improve this analysis and all other studies referring to antibiotic use, a precise cost per prescription (or per patient) due to antibiotic resistance should be identified, so that research can be carried out with more accurate and reliable calculations.

Also, it should be considered that we conducted an analysis from a social point of view, but if the PCT was reimbursed, the tariff might be different from the unit cost estimated in this study and therefore the ICER from the payer’s point of view (i.e. health insurance) might be different. Therefore, we recommended to carry out studies that consider different perspectives when estimating costs.

## Conclusions

Based on the analyses performed, this study shows that even within constrained budgets, i.e. for the WTP range between $2 and $26, procalcitonin point-of-care test has a high probability of being the most cost-effective strategy, showing that for moderate costs, it reduces antibiotic prescriptions considerably. If we consider a higher WTP, UltraPro becomes the strategy with the highest probability of being the most cost-effective.

## Data Availability

Data with details of the trial used to estimate costs are available on request.

## Appendix

### Costs calculation

- Training costs: PCPs received additional training in all groups. These costs were calculated by multiplying the opportunity-cost of working time—based on salary—of PCPs, medical instructors and PCP assistants by the number of hours required for the training.

Costs per patient does not vary for patients in the same treatment arm, as PCP training does not change according to their patient population. We used training times reported in the trial data, PCPs and instructor median wage per hour from Brunner 2019 [22], and medical assistant wages from recommendations made by the local medical society [23]. Since the numbers of patients per PCP differs in each group, and since the training costs vary according to the number of participating PCPs, we took into account the number of PCPs in each respective arm and multiplied it by the average number of patients per PCP among the whole sample. In this way, we obtained an adjusted number of patients per arm, used to divide the training costs to make the groups more comparable. This allowed us to compute a training cost per patient which did not depend on the number of patients recruited in each group.

- Product costs: The devices used for diagnostic testing in the intervention groups incur fixed costs that can be considered as spread among patients. In the same manner as above, we considered the adjusted number of patients. The calculation was made by dividing the purchase costs (price data from Roche Holding AG, Basel, Switzerland, and Koninklijke Philips N.V., Amsterdam, Netherlands) of the devices employed by each PCP—which differ across each group—by their estimated service life in months and adding up the monthly maintenance costs.
- Initial consultation costs: These costs are attributable to the time taken by the PCPs to complete the examination and to the type of examinations performed. We included the physicians’ opportunity cost [22] related to the time required for consultation (length of visits taken from the trial) with the cost of the examinations performed, which vary depending on the arm and the status of the patient. The number of white blood cells, C-reactive protein, procalcitonin, capillary puncture and blood tests performed for each patient were taken from the study and multiplied by standardized Swiss medical tariffs (TARMED) [24].
- Cost of antibiotic prescriptions: These costs depend on whether patients received a prescription for an antibiotic and its corresponding price. The observed amount and typology of prescriptions during the 28-day follow-up were taken from the trial data. The antibiotic prices are those of the most expensive brand according to data from the Federal Office of Public Health (FOPH) [25].
- Cost of other drug prescriptions: We included the costs of all other medication prescribed during the initial consultation (for example paracetamol, non-steroidal anti-inflammatory drugs, inhaled steroids, bronchodilators, antitussives). Each drug indicated in the data was multiplied by its price, based on data from the FOPH [25]. For drugs classified as 'other' by the trial we used the average of the prices of the specified drugs.
- Costs for healthcare treatments: This item includes costs for chest X-rays, hospitalisation, and repeated PCP visits, if any of these events were recorded during the trial. Hospitalisation costs were based on tariffs reimbursed by basic Swiss health insurance [26, 27]. Prices for chest X-rays and repeated PCP visits were taken from TARMED [24]. We assumed that the unit cost of consultation is the same, regardless of the type of doctor and medical centre.
- Productivity loss: These are the costs from patients being partly or completely unable to work. The duration of sick days (days with functional restrictions) is taken from the trial data. We made the following assumptions: patients earn the median monthly Swiss wage (taken from the Federal Statistical Office [28]) and we considered a drop in productivity of one third when patients experience restrictions. We also assumed a day off work in case of any side effects of the antibiotics.
- Antibiotic resistance cost: Overuse of antibiotics leads to the development of treatment-resistant strains of bacteria, which can lead to severe health consequences [29]. Many studies suggest taking into account these indirect costs for society [30–36] but methods are debated and collecting sufficient data is difficult [37]. The idea is to add costs of antibiotic resistance induced by each prescription. One simple option is to divide the total amount of costs due to resistance (more expensive treatments, longer hospitalisation, decreased productivity, increased deaths) by the number of prescriptions per year to get an estimate of the indirect costs per prescription.

We were unable to find data or an estimate of these indirect costs for Switzerland, so we proceeded as follows. As described in Oppong et al. 2016 [38], we took the overall estimated costs—including outpatient care, productivity losses and deaths—caused by antibiotic resistance in the European Union [39, 40] and divided it by the total number of prescriptions [41]. In this way we obtained the average cost among EU countries to be charged per prescription, assuming that the increase in resistance and related costs depend linearly on the number of prescribed antibiotics. Converting the currency, adjusting for inflation (price level as of August 2022) and multiplying by a factor of 2.5—which corresponds to the ratio at current prices of the Swiss GDP per capita to the EU average [42]—we obtained indirect resistance costs of approximately CHF 8.10 ($8.46) per prescription. We then added this amount to the patients who have received at least one prescription.

### Scenario descriptions

Scenario 1 (base-case scenario) does not include hospitalisation costs because, given their small number, the trial was not powered to detect whether the difference in the number of hospitalisations is due to the treatment received or to individual random characteristics. As these costs are extremely high compared to the average, they greatly distort their distribution, leading to extremely high variances. In fact, if hospitalisations are included, the differences between the costs of the three arms are not statistically significant. The effectiveness outcome (the APRs) considered is corrected for the cluster size. Another assumption concerns the way fixed costs are treated. As explained, they are inputted considering a homogeneous number of patients per PCP, so that the three groups are more comparable than dividing training and product costs by the number of patients in the respective arm.

Scenario 2 provides that the antibiotic prescription rates used as denominators of the Incremental cost-effectiveness ratio (ICER) are those directly observed in the study, not corrected for cluster size.

Scenario 3 includes hospitalisation costs. Prescriptions are again those adjusted for cluster size.

Scenario 4 is identical to Scenario 1, except that fixed costs (training and product) have been removed from the analysis. This is to illustrate a potential future situation in which the spread of procalcitonin testing and UltraPro will be facilitated by exploiting economies of scale, which will reduce training and equipment costs.

### Scenario analysis

Below is a comparison of the results of the ICERs in the various scenarios considered.

See Table 3

**Table 3:** Comparison of ICER across different scenarios (95% CI)

Groups comparison	Scenario 1	Scenario 2	Scenario 3	Scenario 4
UltraPro vs usual	4.2 (1.5 to 10.8)	4.2 (1.7 to 6.9)	4.9 (− 2.5 to 16.8)	1.6 (− 0.2 to 4.8)
Procalcitonin vs usual	2.2 (− 0.7 to 7.6)	1.9 (− 0.6 to 4.4)	3.2 (− 5.3 to 15.2)	1.2 (− 0.7 to 4.5)

Even in scenario 3, where average costs rise due to hospitalisations, and where procalcitonin’s ICER rises more than UltraPro’s, it is still more cost-effective to use only procalcitonin testing. However, because of this, the range within which procalcitonin testing is the strategy most likely to be cost-effective is narrower, with the switching value between procalcitonin and UltraPro being about CHF 10 CHF of willingness to pay lower than in scenario 1. In any case, further studies to understand the impact of these diagnostic tools on the number of hospitalisations could be carried out.

Point-of-care procalcitonin testing is likely to become more attractive over time. In scenario 4, where the cost difference drops due to the exclusion of training and product costs, that affect only treatments and not usual care, procalcitonin and UltraPro become even cheaper, with a ratio almost equivalent to that of usual care. This is important to emphasize because, if in the future the training and production costs of the machines can be lowered by involving more doctors and manufacturers, these diagnostic tools will become even cheaper.
